# Programmed death ligand 1 is over-expressed by neutrophils in the blood of patients with active tuberculosis

**DOI:** 10.1002/eji.201141421

**Published:** 2011-04-20

**Authors:** Finlay W McNab, Matthew P R Berry, Christine M Graham, Susannah A A Bloch, Tolu Oni, Katalin A Wilkinson, Robert J Wilkinson, Onn M Kon, Jacques Banchereau, Damien Chaussabel, Anne O'Garra

**Affiliations:** 1 Division of Immunoregulation, MRC National Institute for Medical ResearchLondon, UK; 2 Department of Respiratory Medicine, St Mary's Hospital, Imperial College Healthcare NHS TrustLondon, UK; 3 Institute of Infectious Diseases and Molecular Medicine, University of Cape TownCape Town, South Africa; 4 Division of Medicine, Imperial College London,ondon, UK; 5 Division of Mycobacterial Research, MRC National Institute for Medical ResearchLondon, UK; 6 Roche, Inflammation Discovery, NutleyNJ, USA; 7 INSERM U899, Baylor Institute for Immunology Research-ANRS Center for Human VaccinesDallas, TX, USA; 8 Benaroya Research InstituteSeattle, WA, USA

**Keywords:** Neutrophils, Programmed death ligand 1, Tuberculosis

## Abstract

Tuberculosis (TB), caused by *Mycobacterium tuberculosis* (*Mtb*), remains one of the world's largest infectious disease problems. Despite decades of intensive study, the immune response to *Mtb* is incompletely characterised, reflecting the extremely complex interaction between pathogen and host. Pathways that may alter the balance between host protection and pathogenesis are therefore of great interest. One pathway shown to play a role in the pathogenesis of chronic infections, including TB, is the programmed death-1 (PD-1) pathway. We show here that the expression of the programmed death ligand 1 (PD-L1), which interacts with PD-1, is increased in whole blood from active TB patients compared with whole blood from healthy controls or *Mtb*-exposed individuals, and that expression by neutrophils is largely responsible for this increase.

## Introduction

Infection with *Mycobacterium tuberculosis* (*Mtb*), leading to tuberculosis (TB), remains one of the pre-eminent problems in global health, with an estimated third of the world's population infected with *Mtb* [1]. The majority of infected people remain in a state of ‘latency’, being asymptomatic and non-infectious, as the infection is controlled by the immune response. Nonetheless, a small proportion of infected individuals will suffer active disease or reactivate latent infection resulting in active disease [2].

The immune response to *Mtb* is complex and incompletely understood. Several factors are known to be necessary for control of *Mtb* but there is an incomplete understanding of which host factors are sufficient for successful immune control or which drive disease pathogenesis [2, 3]. Therefore, there is great interest in how immune regulatory systems may modulate the balance between protection and pathogenesis during TB.

Programmed death ligand 1 (PD-L1) (also denoted as CD274 and B7-H1) is an immunomodulatory molecule that acts largely through interaction with the programmed death-1 (PD-1) receptor [4]. PD-1 interacts with its ligands PD-L1 and PD-L2 to deliver inhibitory signals that regulate T-cell and other responses, thus helping to maintain the balance between effective immunity, tolerance and immuno-pathology. PD-L1 is reportedly expressed on a variety of different cell types, including T cells and myeloid cells such as DCs and monocytes [4].

PD-L1/PD-1 interactions can play a role in a diverse array of settings including infectious disease. In an infectious setting, PD-L1/PD-1 interactions are often associated with chronicity, particularly during viral infection [4]. PD-L1 reportedly suppresses T-cell proliferation and effector function, through binding PD-1, most notably on functionally ‘exhausted’ CD8^+^ T cells during chronic viral infections such as mouse models of lymphocytic choriomeningitis virus infection and also on CD4^+^ and CD8^+^ T cells in patients infected with HIV or hepatitis C [5–9].

PD-L1/PD-1 interactions may also play a role in the chronicity of some bacterial infections [10, 11]. T cells from TB patients reportedly express PD-1, and PD-L1 could be induced on T cells stimulated with sonicated H37Rv *Mtb* [12]. Antibodies blocking PD-1/PD-L1/PD-L2 enhanced *Mtb* antigen-specific IFN-γ responses and CD8^+^ T-cell cytotoxicity from peripheral blood and pleural fluid mononuclear cells in vitro [12]. Similar findings have recently been reported for NK cells obtained from pleural fluid and peripheral blood of TB patients [13]. Nevertheless, the expression of PD-1 and its ligands PD-L1 and PD-L2 during TB remains incompletely defined, thus hampering the understanding of how PD-L1/PD-1 may regulate the immune response to *Mtb*.

We have previously conducted whole genome microarray of whole blood from active and latent TB patients and healthy controls and shown a distinct transcriptional signature, comprising both under and over-represented transcripts, for the active TB group [14]. We wished to further investigate these data sets to determine the levels of expression of molecules of the PD-1 pathway in different cell populations in patients with TB.

## Results

### PD-L1 is over-represented in active TB patients' blood and reduced with anti-mycobacterial treatment

**Figure 1:**
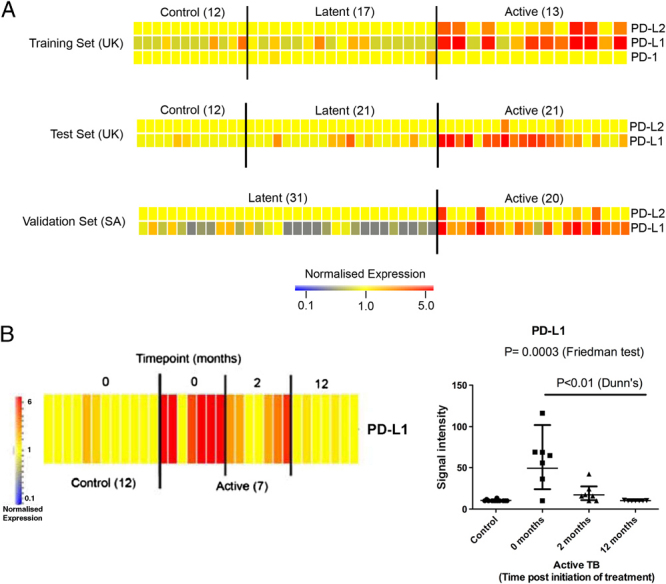
PD-1, PD-L1 and PD-L2 gene expression in active TB and during anti-mycobacterial treatment. (A) PD-1, PD-L1 and PD-L2 gene transcript abundance in whole blood samples from active TB patients, latent TB patients and healthy controls. (B) PD-L1 transcript abundance in whole blood samples from seven active TB patients at the time of diagnosis and at 2 and 12 months post-initiation of anti-mycobacterial treatment, compared with healthy controls, shown by heatmap and graph. Gene abundance is shown normalised to the median of all samples. Each row of the heatmaps represents an individual gene and each column an individual participant. The relative abundance of transcripts is indicated by a colour scale (red, high; yellow, median; blue, low). Each point on the graph represents one subject and the line and error bars the mean and 95% confidence interval. Statistical significance was analysed using (A) Kruskal–Wallis one-way ANOVA with Benjamini–Hochberg multiple testing correction (PD-L1: active versus latent and control, training set *p*=0.0043, test set *p*=0.000521, validation set *p*<0.0001) and (B) Friedman test with Dunn's post hoc analysis.

Initially, we investigated the expression of transcripts for the main molecules involved in the PD-1 pathway, namely PD-1, PD-L1 and PD-L2, in whole blood from active and latent TB patients and healthy controls (Fig. 1A). PD-L1 was significantly over-represented in whole blood from active TB patients as compared with healthy controls and latent TB patients in three independent cohorts of patients (Fig. 1A); two recruited in London (Training and Test sets, *p*=0.0043 and 0.000521, respectively) and one from a TB endemic area (South Africa; validation set, *p*<0.0001). The abundance of PD-L2 was increased in some active TB patients, particularly in the training set; however, PD-L2 levels overall were not significantly different in all three cohorts (Fig. 1A). PD-1 levels were not increased in active TB patients compared with latent TB patients and healthy controls in all three cohorts (Fig. 1A and data not shown).

In addition, we investigated how PD-L1 levels were affected during successful treatment of disease with anti-mycobacterial chemotherapy. Seven active TB patients were tracked longitudinally during anti-mycobacterial treatment and PD-L1 levels assessed at time of diagnosis and at 2 and 12 months after initiation of treatment. The over-representation of PD-L1 in blood of active TB patients seen at the time of diagnosis was significantly decreased with treatment (Fig. 1B). One patient did not have increased levels of PD-L1 at time of diagnosis; however, upon further clinical analysis, this patient showed no radiographic disease [14]. Although some active TB patients still showed elevated levels of PD-L1 transcripts at 2 months post-initiation of treatment, there was no difference in PD-L1 transcript levels in patients compared with healthy controls after completing successful therapy (Fig. 1B).

Together, these findings suggest that the presence of PD-L1 in the blood may be related to pathology and failure to control disease during TB. In this regard, our findings would be consistent with reports both in murine models and human studies of chronic viral and bacterial infections [5–13, 15, 16].

### PD-L1 is upregulated on the cell surface of blood leucocytes from active TB patients

**Figure 2:**
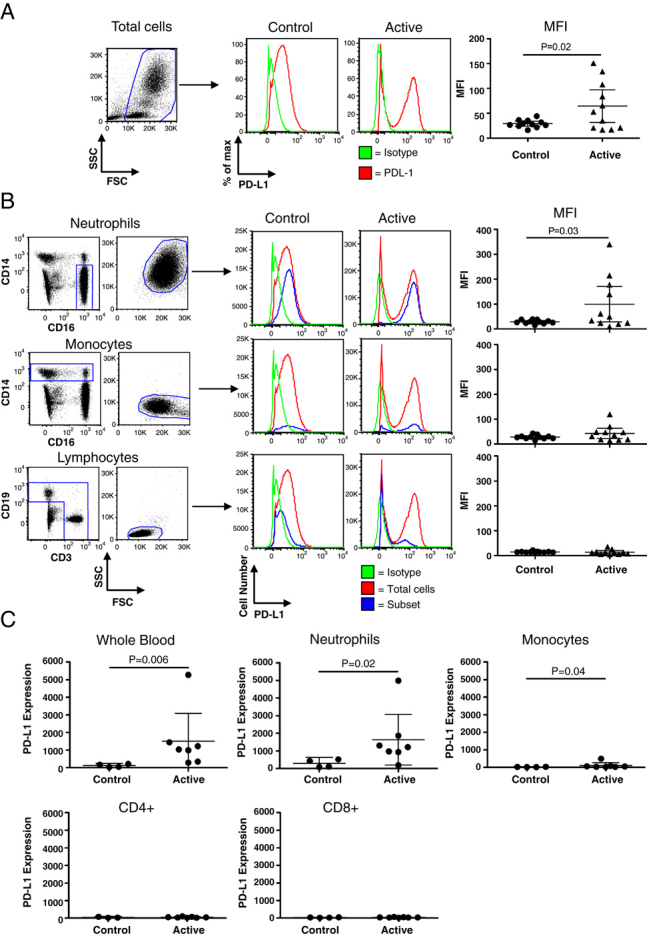
Levels of PD-L1 in different cell populations from whole blood. (A) Whole leucocytes from 11 healthy controls and 11 active TB patients were stained with anti-PD-L1 antibodies and expression levels determined. An example of a healthy control and active TB patient are shown. (B) Gates were then set on cells representing neutrophils, monocytes and lymphocytes, which were assessed for PD-L1 expression. Different cell subpopulations are shown in blue; total leucocytes in red and isotype control (total cells) in green. Isotype levels on separated cells were not different to levels on total cells (data not shown). Neutrophils were defined as CD16^+^CD14^–^ forward scatter (FSC) high, side scatter (SSC) high, monocytes as CD14^+^ FSC intermediate SSC intermediate and lymphocytes as CD3^+^CD19^+^ FSC low, SSC low. Graphs for (A) and (B) show pooled data of the geometric mean fluorescence intensity of PD-L1 on each subset for all subjects. Each symbol represents one subject; lines and error bars the mean and 95% confidence interval. Statistical significance was analysed using unpaired *t*-test. (C) Whole blood, separated neutrophils, monocytes and CD4^+^ and CD8^+^ cells from seven active TB patients and four healthy controls were analysed by Nanostring technology for PD-L1 gene expression. Graphs show pooled data of PD-L1 expression for each subset. Each symbol represents one subject; lines and error bars the mean and 95% confidence interval. Statistical significance was analysed using Mann–Whitney *t*-test.

To elucidate whether the over-representation of PD-L1 in blood of active TB patients resulted from a general increase in numbers of cells expressing PD-L1 or from increased expression of PD-L1 by a particular cell type, flow cytometric analysis of blood from test set active TB patients and healthy controls was conducted. There was variation between patients, reflected by spread in MFI of PD-L1 expression (Fig. 2A), likely representing the heterogeneity of TB patients, extent of disease and the natural variability in the human immune response to *Mtb*. However, overall expression of PD-L1 on whole blood leucocytes from patients with active TB was significantly increased compared with blood leucocytes from healthy controls (Fig. 2A; Supporting Information Fig. 1A), in line with the microarray findings.

### Neutrophils are the predominant cell type expressing PD-L1 in blood of active TB patients

Since PD-L1 expression was increased on a proportion but not all leucocytes from blood of active TB patients, we were interested to know if a discrete cell population was expressing PD-L1. Further flow cytometric analysis revealed that PD-L1 was most highly expressed on cells that were consistent with CD16^+^ neutrophils, whereas few cells consistent with CD14^+^ monocytes or CD3^+^ or CD19^+^ lymphocytes from active TB patients expressed PD-L1 (Fig. 2B; Supporting Information Fig. 1B). Again, relatively low levels of expression of PD-L1 were found on all analysed cell types from the blood of healthy controls (Fig. 2B; Supporting Information Fig. 1B).

To confirm the increased level of neutrophil-specific PD-L1 expression in active TB patients, neutrophils, monocytes, CD4^+^ and CD8^+^ T cells from blood of active TB patients and healthy controls were separated using magnetic beads, and the levels of PD-L1 transcripts determined using Nanostring nCounter technology (Fig. 2C). This technology allows capture and counting of individual mRNA transcripts in a complex mixture and has similar sensitivity to real-time PCR [17]. Nanostring analysis of purified cell populations showed that purified neutrophils from active TB patients expressed significantly higher levels of PD-L1 transcripts than purified neutrophils from healthy controls (Fig. 2C), and this increased expression on neutrophils appeared to account for the total increase in PD-L1 expression in whole blood. Monocytes from active TB patients also had slightly higher expression of PD-L1 than monocytes from healthy controls, and although levels were significant they were much lower than in whole blood or neutrophils. There was no increased PD-L1 expression in CD4^+^ and CD8^+^ T cells above controls (Fig. 2C). Thus, neutrophils appear to be the predominant cell type over-expressing PD-L1 in the blood of active TB patients.

To our knowledge, this is the first report of PD-L1 expression by neutrophils during in vivo infection, although a recent report has shown that treatment of neutrophils with IFN-γ and GM-CSF upregulated PD-L1 [18] and an earlier report showed that LPS stimulation could upregulate PD-L1 on neutrophils [19]. We have previously found that neutrophils from active TB patients display an IFN-inducible signature that contained gene transcripts involved in both type I and type II IFN signalling [14]. Our results are therefore consistent with these earlier findings [18] but also suggest that type I IFN may play a role in PD-L1 upregulation on neutrophils, particularly as type I IFN reportedly upregulates PD-L1 on other cell types [20, 21].

How PD-L1 expression on neutrophils modulates the immune response during TB remains to be elucidated; however, it is possible that PD-L1 expression acts as a mechanism for limiting neutrophil-mediated immunopathology. This is in accordance with recent findings that PD-1^−/−^ mice infected with *Mtb* have severe lung pathology and succumb to disease much earlier than WT mice [22]. This was associated with a dramatic increase in neutrophils in the lungs [22].

## Concluding remarks

In this study we show that the immunomodulatory ligand PD-L1 is over-represented in the blood of patients with active TB, that this over-representation is largely driven by neutrophil expression of PD-L1 and is diminished by successful therapy. This is consistent with an association of PD-L1 with failure to control disease and pathology. Whether PD-1/PD-L1 interactions act to suppress protective immunity during TB or as a mechanism for controlling neutrophil-mediated immunopathology will be important to elucidate. The majority of studies of PD1/PD-L1 interaction during chronic viral infection point to this pathway acting to suppress protective CD8^+^ T-cell responses [5–8]. PD-1/PD-L1 interaction may also therefore suppress protective responses during TB [12, 13]. Indeed, there is evidence that the PD-1/PD-L1 pathway suppresses *Mtb*-specific IFN-γ production and cytotoxicity by T cells as well as IFN-γ production and cytotoxicity by NK cells from peripheral blood and pleural fluid mononuclear cells from TB patients [12, 13]. Whether neutrophils expressing PD-L1 are able to suppress these protective responses in active TB patients would be important to investigate.

However, there is growing evidence that in intracellular bacterial infections, where protection is largely mediated by CD4^+^ T cells, the role of the PD-1/PD-L1 pathway may be less straightforward. Two models of *Listeria* infection demonstrated that abrogation of PD-L1 signalling during infection led to reduced antigen-specific T-cell responses, inhibition of key effector molecules, increased bacterial loads and increased mortality [23, 24]. In addition, recent reports have shown that PD-1^−/−^ mice infected with *Mtb* also have significantly higher bacterial loads in the lung, increased lung pathology and earlier mortality than wild-type mice [22, 25]. Large increases in pro-inflammatory cytokines and an increase in neutrophils but a decrease in T cells in the lungs were also observed [22]. Another report has shown that PD-1 expressing CD4^+^ T cells during murine *Mtb* infection are not analogous to the functionally exhausted PD-1-expressing CD8^+^ T cells seen during chronic viral infections but rather are a highly proliferating, cytokine-producing population [26]. The effect of PD-1/PD-L1 interactions on innate and adaptive immunity during bacterial infection is therefore likely to be more complex than at first thought and this may complicate their use as a potential therapeutic target.

## Materials and methods

### Patient cohorts and healthy controls, sample collection and processing for microarray

This study was approved by the local Research Ethics Committees at St Mary's Hospital London, UK (REC 06/Q0403/128) and University of Cape Town, Cape Town, Republic of South Africa (REC012/2007). All participants were over 18 years of age and gave written informed consent. See Berry et al. [14] for further details.

Blood (3 mL) was collected into Tempus tubes (Applied Biosystems, CA, USA) and stored between −20 and −80°C. All patients were sampled before treatment. The diagnosis of active TB was confirmed by positive culture for *Mtb*. Latent TB patients were asymptomatic household contacts of active TB patients or new entrants from endemic countries, defined by a positive tuberculin-skin test (TST) (London) and a positive *Mtb* antigen-specific Interferon-γ Release Assay (London and South Africa; QuantiFERON Gold In-Tube Assay, Cellestis). Ethnically matched healthy controls were recruited in London and were negative for all the above criteria. The cohorts were independently recruited and sampled. RNA preparation, all processing and analysis of samples from the three cohorts were performed independently. Genome-wide transcriptional profiles from the blood of active TB patients, latent TB patients and healthy controls were generated using Illumina HT12 V3 beadarrays as previously described [14].

### Micorarray data analysis

Illumina BeadStudio v2 software was utilised to subtract background and scale average signal intensity for each sample to the global average signal intensity for all samples. Further normalisation was performed using the gene expression analysis software program, GeneSpring GX, version 7.1.3 (Agilent Technologies, Santa Clara, CA, USA). All signal intensity values <10 were set equal to 10. Per-gene normalisation was then applied by dividing the signal intensity of each probe in each sample by the median intensity for that probe across all samples.

### Flow cytometry

Antibody staining of whole blood for flow cytometry was performed as previously described [14]. Samples were run on a Beckman Coulter CyAn using Summit Software Version 3.02. All antibodies were purchased from BD Pharmingen or Caltag Laboratories (Invitrogen). Analysis was carried out using FlowJo Version 8.7.3 for Macintosh (Tree Star).

### Cell separation and RNA preparation

Cell separations and subsequent RNA preparation were performed as previously described [14]. Samples were then processed using Nanostring technology and analysed by the GeneSpring GX7.3.1 software.

### Gene expression analysis using Nanostring technology

The nCounter Analysis System (Nanostring) [17] was used to validate gene expression seen previously by microarray analysis. mRNA was detected using a custom-made nCounter Reporter probe set (84 transcripts). A total of 100 ng total RNA was hybridised overnight at 65°C in a thermocycler to reporter and capture probe sets to form target/probe complexes. Following hybridisation, excess probes were washed away on the nCounter Prep Station. Colour-coded barcodes on the reporter probe were read on an nCounter Digital Analyser to give a quantitative measure of RNA. Using Microsoft Excel, samples signal values were scaled and normalised to the positive control spikes, and then background subtracted. All signal intensity values <10 were set equal to 10.

## Abbreviations

*Mtb*: *Mycobacterium tuberculosis*
PD-1: programmed death-1
PD-L1: programmed death ligand 1
PD-L2: programmed death ligand 2
TB: tuberculosis
