# Supplementary material for: Programmed death ligand 1 is over-expressed by neutrophils in the blood of patients with active tuberculosis
Source: Eur J Immunol. 2011 Apr 20;41(7):1941–7. doi: 10.1002/eji.201141421 (PMC3179592; doi:10.1002/eji.201141421)
Supplement: Supplementary file 1 [file eji0041-1941-SD1.pdf]

European Journal of Immunology

Supporting Information

for

DOI 10.1002/eji.201141421

Programmed death ligand 1 is over-expressed by neutrophils in the blood of patients with active tuberculosis

Finlay W. McNab, Matthew P. R. Berry, Christine M. Graham, Susannah A. A. Bloch, Tolu Oni, Katalin A. Wilkinson,
Robert J. Wilkinson, Onn M. Kon, Jacques Banchereau, Damien Chaussabel and Anne O'Garra

© 2011 WILEY-VCH Verlag GmbH & Co. KGaA, Weinheim
